# Investigator Initiated Clinical Trials (IICTs): A Systematic Search in Registries to Compare the Czech Republic and Portugal in Terms of Funding Policies and Scientific Outcomes

**DOI:** 10.1007/s43441-021-00293-w

**Published:** 2021-05-18

**Authors:** C. Madeira, L. Hořavová, F. dos Santos, J. R. Batuca, K. Nebeska, L. Součková, C. Kubiak, J. Demotes, R. Demlová, E. C. Monteiro

**Affiliations:** 1 European Clinical Research Infrastructure Network (ECRIN), Paris, France; 2 Portuguese Clinical Research Infrastructure Network (PtCRIN), Lisbon, Portugal; 3 Chronic Diseases Research Centre (CEDOC), NOVA Medical School, Universidade NOVA de Lisboa, Lisbon, Portugal; 4 Comprehensive Health Research Centre (CHRC), Lisbon, Portugal; 5 Department of Pharmacology, Faculty of Medicine, Masaryk University, Brno, Czech Republic; 6 University Hospital St. Anne’s Brno – International Clinical Research Centre, Brno, Czech Republic; 7 Department of Clinical Trials, Masaryk Memorial Cancer Institute Brno, Brno, Czech Republic; 8 Department of Applied Pharmacy, Faculty of Pharmacy, University of Veterinary and Pharmaceutical Sciences Brno, Brno, Czech Republic

**Keywords:** Investigator initiated clinical trials, Funding, Clinical trials registry, Clinical research, Clinical trial, Clinical research outcome

## Abstract

**Objectives:**

Clinical trials provide one of the highest levels of evidence to support medical practice. Investigator initiated clinical trials (IICTs) answer relevant questions in clinical practice that may not be addressed by industry. For the first time, two European Countries are compared in terms of IICTs, respective funders and publications, envisaging to inspire others to use similar indicators to assess clinical research outcomes.

**Methods:**

A retrospective systematic search of registered IICTs from 2004 to 2017, using four clinical trials registries was carried out in two European countries with similar population, *GDP, HDI* and medical schools but with different governmental models to fund clinical research. Each IICT was screened for sponsors, funders, type of intervention and associated publications, once completed.

**Results:**

IICTs involving the Czech Republic and Portugal were *n* = 439 (42% with hospitals as sponsors) and *n* = 328 (47% with universities as sponsors), respectively. The Czech Republic and Portuguese funding agencies supported respectively 61 and 27 IICTs. Among these, trials with medicinal products represent 52% in Czech Republic and 4% in Portugal. In the first, a higher percentage of IICTs’ publications in high impact factor journals with national investigators as authors was observed, when compared to Portugal (75% vs 15%).

**Conclusion:**

The better performance in clinical research by Czech Republic might be related to the existence of specific and periodic funding for clinical research, although further data are still needed to confirm this relationship. In upcoming years, the indicators used herein might be useful to tracking clinical research outcomes in these and other European countries.

**Supplementary Information:**

The online version contains supplementary material available at 10.1007/s43441-021-00293-w.

## Introduction

In the last decades, clinical research played a crucial role on increasing the medical knowledge for the prevention, diagnostic, treatment and cure of diseases. Clinical trials provide one of the highest levels of evidence to support medical practice and might be initiated by the industry or by clinical investigators. The latter, commonly called investigator-initiated clinical trials (IICTs), are interventional studies using medicinal products, medical devices, surgery techniques, behavior testing or several types of procedures. These trials are pivotal to generate relevant and unbiased data decisive for the implementation of new therapies and better-informed decisions by health regulators [1]. However, the importance and relevance of IICTs rely heavily on its independence and impartiality, which in turn is directly linked to the funding source. Some IICTs are funded by industry, which can introduce a bias in the outcome of these studies. In fact, a systematic review of 1140 studies demonstrated that industry-sponsored clinical trials were significantly more likely to reach conclusions favorable to the sponsors than those who were not industry-sponsored trials [2].

Therefore, public national funding agencies have a critical role on supporting fully independent clinical research with the sole purpose of benefiting the large patients´ population through the improvement of medical knowledge. Public funding of IICTs can also play an important role on addressing important clinical questions that remain unresolved because they are not addressed in industry-funded trials. Crowe and colleagues showed that there is an important mismatch on clinical research priorities identified by the patients and the clinicians, which in part is affected by the commercial aspect of clinical research [3].

The unbalanced ratio between private and public funding of IICTs (and its impact on national health systems through the definition of public policies for the treatment of diseases) is particularly relevant in countries with negligible expenditure in this area, which is usually associated to limited funds for the promotion of clinical research. It is important to generate significant scientific data to be able to provide better and informed guidance for the funding of IICTs by the national public funding agencies.

The Czech Republic and Portugal have similar population numbers (10.6 million and 10.3 million, respectively), GDP per capita (€14.8 thousand and €16.9 thousand, respectively), Human Development Index (2017: 0.896 and 0.858, respectively) and number of Medical Schools (*n* = 9 and *n* = 8, respectively), which makes them comparable countries for such study. In a previous publication, and considering only clinical trial registries in the European Clinical Trials Registry (EU-CTR) it was shown that Czech Republic and Portugal are both below the average among all European countries in what regards the percentage of IICT/Total CT (5% and 8%, respectively) [4].

Both countries are members of the European Union and thus have access to the same funding schemes and also operating under to the same health regulatory environment (supervised by the European Medicines Agency). Additionally, both countries are members of the European Clinical Research Infrastructure Network (ECRIN-ERIC), which is a public, non-profit organisation committed in supporting and facilitating multinational independent clinical trials in Europe.

Currently, the Czech Clinical Research Infrastructure Network (CZECRIN) aims its activities to bring together academic research institutes, scientists and policymakers in discussion on issues related, particularly public funding of the clinical research in the Czech Republic. CZECRIN’s specific goal is to promote and support academic research on the national basis and the integration of national investigators in multinational clinical studies.

The Portuguese Clinical Research Infrastructure Network (PtCRIN) has been developing several national studies where the status of IICTs in Portugal were assessed [4, 5]. Despite the relevance of such data to define national strategies for this area, the impact of such analysis can be maximized through a comprehensive benchmarking with other European countries, particularly the ones with similar characteristics. Therefore, intending to perform a comprehensive assessment of the current situation, this study analyzed the type of registered IICTs in the Czech Republic and in Portugal and for the first time unveiled the differences in terms of funding policies and scientific outcome.

## Methods

We identified past or current IICTs registered and starting in two European countries (Czech Republic and Portugal) from 01/01/2004 to 31/12/2017 to identify detailed studies´ characteristics such as the funding sources and the respective scientific impact. This period of time starts in the year after which the International Committee of Medical Journal Editors’ implementation (ICMJE), policy required the registration of clinical trials as a prerequisite for consideration for publication (2004) until the end of 2017. A separate search in four clinical trial registries (CTRs) was performed for each country: ClinicalTrials.gov, European Clinical Trials Registry (EU-CTR), International Standard Randomised Controlled Trial Number (ISRCTN) registry, and the Australian New Zealand Clinical Trials Registry (ANZCTR) by selecting trials registered and with recruitment sites in Czech Republic or Portugal. These CTRs encompass 81% of the registrations uploaded to the International Clinical Trials Registry Platform (ICTRP) from WHO, according to the information provided by others [6]. Furthermore, we collected and compared the national public policies implemented by the Portuguese and Czech governments to promote clinical research during this timeframe.

### Search Methodology for Clinical Trials Identification in Each Database

The inclusion criteria were: clinical trial starting from 01/01/2004 until 31/12/2017; non-commercial sponsor; sponsor or recruiting sites in Portugal or in Czech Republic. The following exclusion criteria were used: industry sponsored trials; starting date before 01/01/2004; sponsor or recruitment site not involving the Czech Republic or Portugal; non-intervention trials.

Step 1: The search in the EU-CTR allowed to include as general term “non-commercial” therefore eliminating the commercial studies. The timeframe selected was from 01/01/2004 until 31/12/2017 as well as the countries, the Czech Republic or Portugal. No other requirement in the advanced search field was selected.

Step 2: As the discrimination between commercial and non-commercial clinical trial is not possible at the ClinicalTrials.gov platform, an advanced search was performed. The IICTs were extracted by selecting the predefined options: “Interventional studies”, the above-mentioned time period, each one of the countries, restricting to: NIH (U.S. National Institutes of Health), Other U.S. Federal agency, all others (individuals, universities, organizations) and Industry were chosen.

Step 3: Registrations in the BiomedCentral CTR are associated with ISRCTN. In this CTR it was not possible to refine the search with the exception of selecting the Czech Republic or Portugal as the recruiting country. Trials initiated before or after the referred timeframe were discarded.

Step 4: In the ANZCTR, we performed the search in the selected timeframe, using the Czech Republic or Portugal as the recruiting country. Registrations in this CTR have the initial code ACTRN.

The search results were reviewed individually by two independent experts in each national team to confirm the compliance with the inclusion and exclusion criteria.

### Data Extraction, Duplicates and Complementary Information

Step 5: Information about trial identification (Trial ID) number (main and secondary), recruitment status, sponsor name and country, trial title, trial phase (when applicable), type of intervention, therapeutic area, type of funding and publications of the completed studies, were extracted manually and independently from the registered records (from 1/03/2018 to 31/08/2018), gathered and organized in an Excel sheet for each country. No restrictions on each of the referred information was applied. Forty percent of the records were double-checked by two individuals from each team and discussed until a consensus was reached.

Step 6: Duplicates were identified in each database, when secondary IDs were provided or when the sponsor and the study title were the same. The same study registered in different CTRs was considered duplicated and removed at this point of the search. When complementary information was provided in different registrations about the same study it was added to the respective entry in the working Excel sheet. None of the studies identified had recruitment sites in both countries, so no clinical trial is considered in both databases.

Sponsors were coded as: Disease-Specific Organization (Disease associations or research institutes dedicated to a specific therapeutic area), Foundation (Research Institutes or Health Institutions legally constituted as foundations), Hospital, Research Institutes (non-specific therapeutic area), University, and others (Private health clinic, Funder Agency, acting as sponsor). In the four CTRs, the funding source was identified in different fields. Both in ISRCTN and ANZCTR there is a specific field entitled “Funder”. In Clinicaltrials.gov CTR the funders were identified from the field “Sponsor and Collaborators”. In EU-CTR the funder was identified in the field “sources of monetary support “. All the funders identified in these CTRs were classified as: (i) Public organizations (non-profit organization such as public institutions, funding agencies, disease specific organizations); (ii) Private organization (for-profit organization—Industry); (iii) Both, when the funding is provided by the industry and one or more public organizations; (iv) Not Indicated, when the information was not provided in any registry or in the publication.

When only the sponsor was mentioned in the ClinicalTrials.gov registry, we considered it as the funder. The support of funding agencies was perceived through secondary IDs where the code of the grant agreement is in some cases added. Exclusive funding for PhD or Post-Doctoral grants was not considered.

### Search of Publications of Completed IICTs

Step 7: Information regarding the publications of the registered and completed IICTs were manually and independently screened. The Trial ID was used to search abstracts of journals indexed in Medline (using PubMed) as well as in the four CTRs. By the principal investigator´s name was possible to identify publications, when no paper was found with the previous strategies. In some publications, the Trial ID was not included in the abstract, which rendered the search more difficult. All completed trials and published until 31/12/2017 were considered for further analysis. Subsequently, screening of each publication was performed to complete the information about the funding sources of IICTs to identify any other relevant information that was not complete in CTRs. The journal’s impact factor was obtained from Web of Science, Research Gate or Bioxbio.com, considering this order when different impact factors for the year of publication were found for the same journal. The citation number since the year of publication up to 1 year after the paper was published was assessed through Pubmed metrics. Around 40% of the data were mutually exchanged between Czech and Portuguese team and double checked by two independent reviewers.

## Results

A total of 3496 and 1427 registrations were found in the four CTRs involving the Czech Republic and Portuguese institutions, respectively: ClinicalTrials.gov, EU-CTR, ISRCTN, and ANZCTR. Clinical trials were screened to isolate those with a non-commercial sponsor, recruiting sites in Czech Republic or Portugal, and a start date between 01/01/2004 and 31/12/2017.

### Identification of Non-commercial Studies

**Figure 1:**
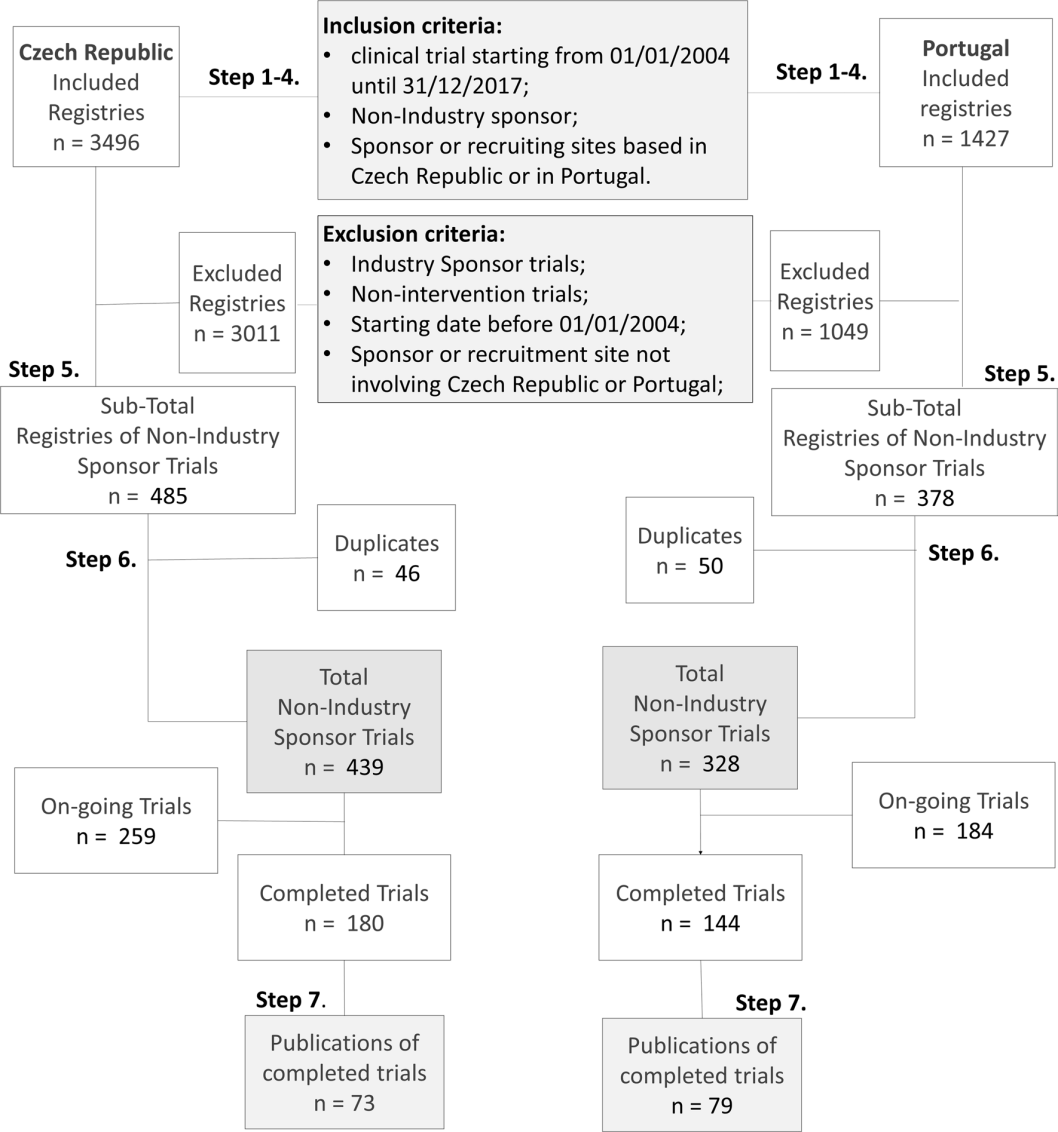
Flowchart Showing the Systematic Search of Non-commercial Clinical Trials (i.e. IICTs), Involving the Czech Republic and Portuguese Institutions Recruiting Participants. The search was performed in four clinical trial registries, CTRs (EU-CTR, Clinicaltrials.gov, ISRCTN and ANZCTR). Studies starting from 01/01/2004 until 31/12/2017 were identified in each of the databases separately (Steps 1–4). After discarding commercial trials all remaining studies were gathered in one Excel sheet for each country (Step 5). Duplicate studies were also discarded, and the final number of trials was cleaned and harmonized (Step 6). Further details were collected from all the databases, including the identification of completed studies. Publications published until December 2017, with results from completed studies were identified (Step 7).

The total number of trials for Czech Republic and Portugal, identified in all databases is respectively 3496 and 1427 as shown in Fig. 1. After discarding industry sponsored trials, non-interventional studies, or those with no recruiting sites in the Czech Republic or Portugal, 485 and 378 non-commercial trials (i.e. IICTs) were respectively considered eligible from all the screened databases. The same trial registered in different CTRs was separated and considered as duplicate. After discarding duplicates, a total number of 439 IICTs were identified involving the Czech Republic whereas for Portugal 328 trials were found. Forty-one percent (*n* = 180/439) and 44% (*n* = 144/328) of the studies were already completed and from those 41% (*n* = 73/180) and 55% (*n* = 79/144) were published, in the Czech Republic and Portugal, respectively.

### Characteristics of IICTs in the Czech Republic and in Portugal

**Figure 2:**
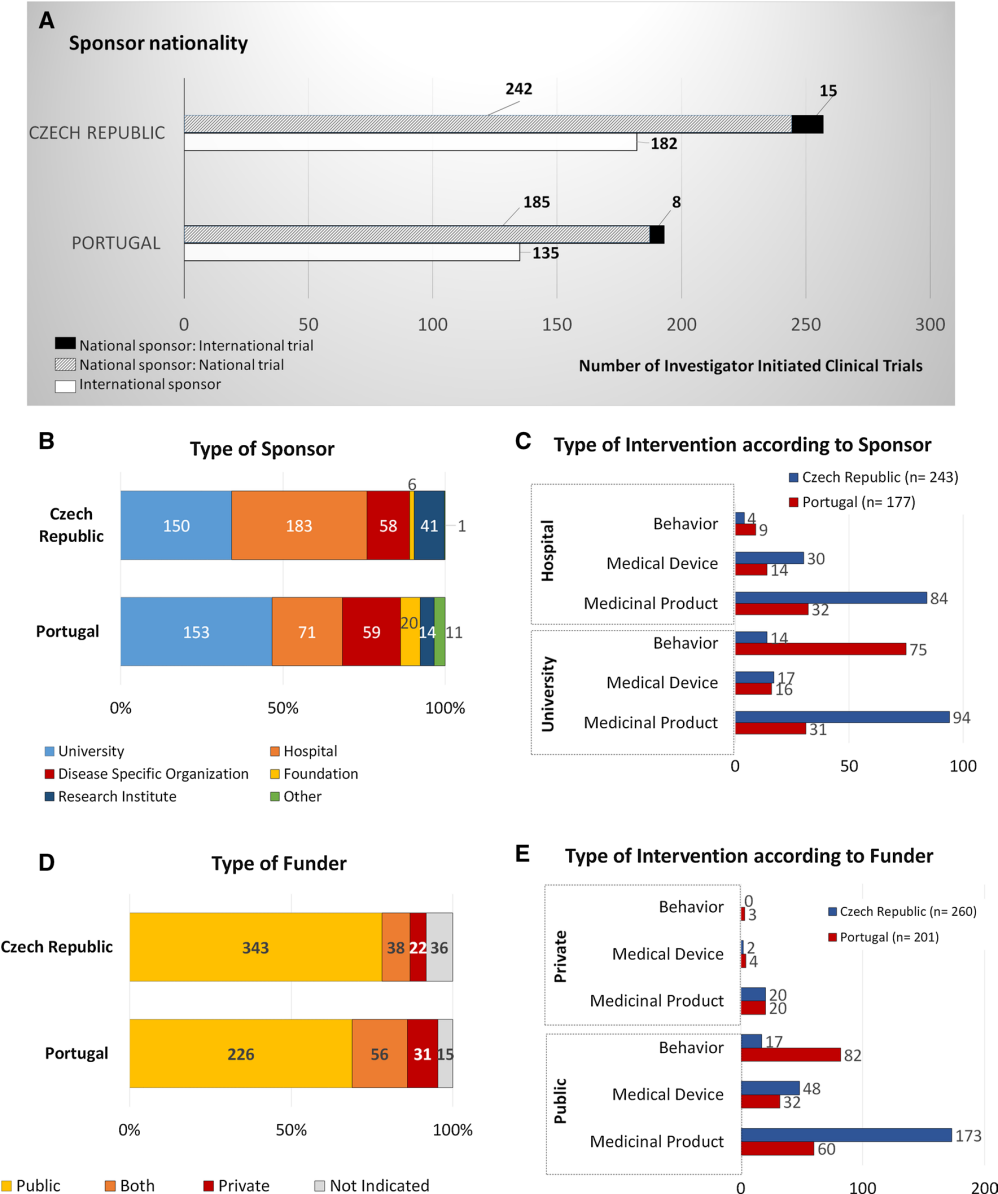
Characteristics of IICTs in the Czech Republic and in Portugal. Number of trials according to the sponsor (national vs international) and to the involvement of other countries in national sponsored trials (national vs multinational trial) (**a**); Percentage and number of trials according to the type of sponsor organization (**b**) and number of trials according to the type of most frequent interventions sponsored by universities or hospitals (*n* = 243 out of 333 for the Czech Republic and *n* = 177 out of 224 for Portugal) (**c**); Percentage and number of trials according to the type of funder (**d**) and number of trials according to the type of most frequent intervention funded by public or private organizations (*n* = 260 out of 365 for the Czech Republic and *n* = 201 out of 257 for Portugal) (**e**).

In both countries, most of IICTs has a national sponsor and were performed in the respective country (Fig. 2a). These IICTs, with national sponsors are those initiated by investigators from each of these countries. Only a low percentage of IICTs with national sponsor are also multinational trials (implemented in other countries)—6% in the Czech Republic (*n* = 15/257) and 4% in Portugal (*n* = 8/193).

Regarding the international trials which are those initiated by investigators from other countries and conducted in The Czech Republic or in Portugal, both countries have also the same percentage (41%; *n* = 182/439 and *n* = 135/328) (Fig. 2a).

In the Czech Republic, most trials´ sponsors (42%; *n* = 183/439) were hospitals while in Portugal, universities assume the sponsorship in higher number of trials when compared to the other types of organizations (47%; *n* = 153/328) (Fig. 2b).

Figure 2c shows that trials with hospitals as the sponsor were testing investigational medicinal products (IMPs) (46% (*n* = 84/183), and 45% (*n* = 32/71), respectively in the Czech Republic and Portugal), medical devices (16% (*n* = 30/183), and 20% (*n* = 14/71), respectively in Czech Republic and Portugal) and behavior (2% (*n* = 4/183), and 13% (*n* = 9/71), respectively in the Czech Republic and Portugal). Regarding all trials sponsored by universities, 62% (*n* = 94/150) test IMPs in the Czech Republic, whereas in Portugal, the largest group by intervention type, 49% (*n* = 75/153), focuses on behavior (Fig. 2b, c). The percentage of trials with IMPs in phase III trials is quite similar in both countries: 32%, (*n* = 82/258) in the Czech Republic and 39% (*n* = 54/136) in Portugal (Supplementary Information 1 (S1), Fig. 1a, a1). Additionally, the two most focused therapeutic areas of the interventional studies are the same: oncology and cardiovascular diseases, followed by gastroenterology in the Czech Republic and musculoskeletal disorders in Portugal (S1, Fig. 1b, c).

IICTs in both countries are mainly funded by public organizations (Fig. 2d) such as universities, hospitals, disease specific organizations, foundations and funding agencies: 78% (*n* = 343/439) in the Czech Republic and 69% (*n* = 226/328) in Portugal. Considering all types of interventions funded by public organizations, 50% (*n* = 173/343) are trials with IMPs in the Czech Republic and only 27% (*n* = 60/226) in Portugal. In the latter, most trials funded by public organizations, 36% (*n* = 82/226), are behavior-based (Fig. 2d, e). Details about each registry are provided as Supplementary Information 2 (S2).

### Clinical Trials Funded by National and International Funding Agencies

**Figure 3:**
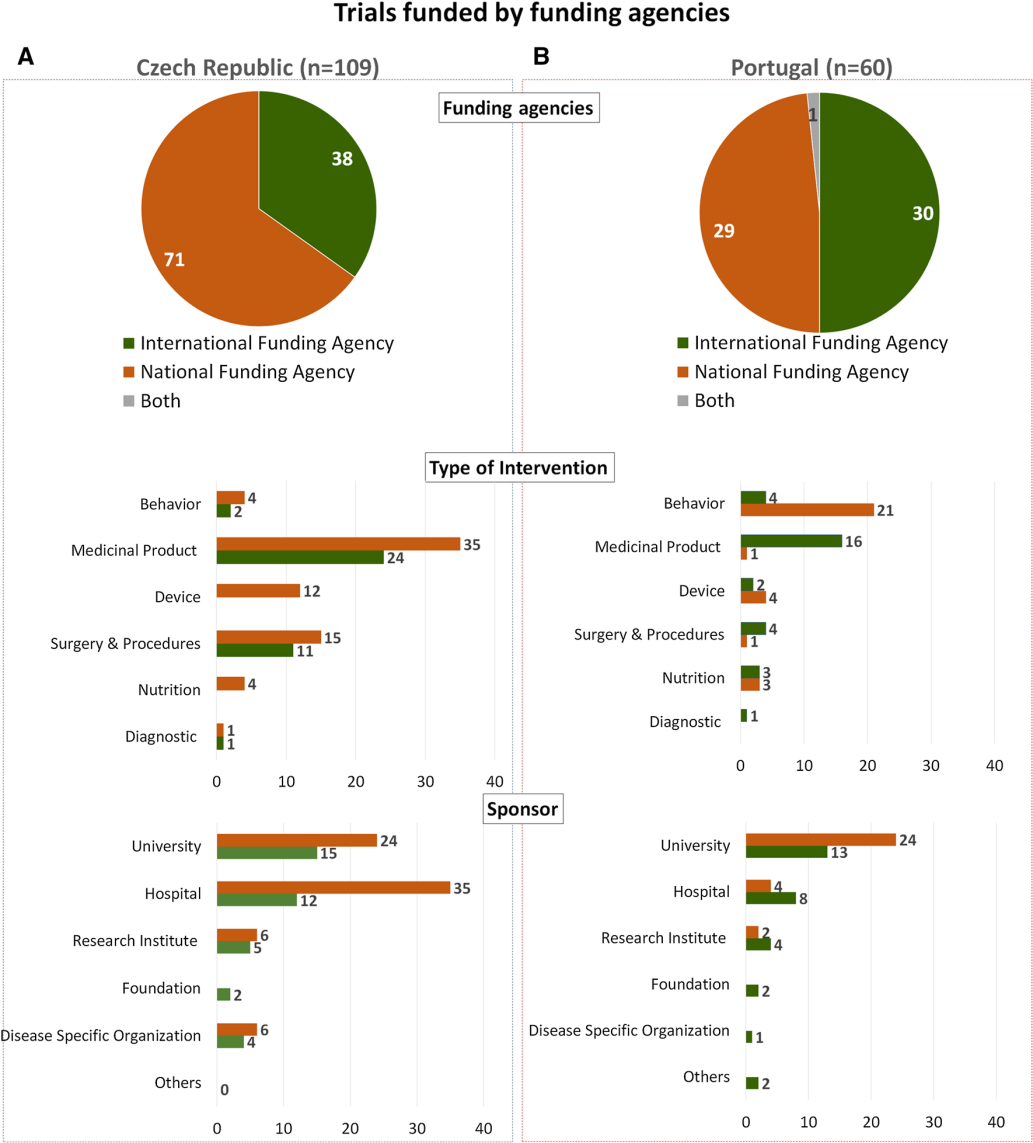
Characteristics of IICTs Funded by Funding Agencies. Number of trials funded with national or international funding agencies in the Czech Republic (**a**) and in Portugal (**b**), according to the type of intervention and sponsor.

One of the greatest public funders of the IICTs in both countries were funding agencies (national and international) corresponding to 29% (*n* = 109/381) and 21% (*n* = 60/282) of the public funding in the Czech Republic and in Portugal, respectively. The total number of trials indicated above (381 and 282) refers to those funded by public organizations alone or together with private institutions (Fig. 2d: Public and Both). From the 109 IICTS trials funded by governmental agencies in the Czech Republic 65% (*n* = 71/109) of the trials were funded by national funding grant agencies while the remaining 35% (*n* = 38/109) IICTs were funded by international counterparts (Fig. 3a). Figure 3a shows that in this country both national and international agencies mainly fund trials with IMPs. Among the 61 trials funded by the Czech Health Research Council (Table 1), 32 (52%, *n* = 32/61) are testing medicinal products (data not shown).

**Table 1:** National and International Funding Agencies and Programs in Czech Republic in and Portugal and Respective Number of Funded Trials. (Nº = Number of Clinical Trials Funded by the Respective Funding Organization/Program).

Funders		Czech Republic	Portugal
National Funding Agencies/Funding Programs	Internal Grant Agency of the Czech Ministry of Health (IGA until 2014); Currently AZV (Czech Health Research Council)	61	–
	Charles University Research Fund	10	–
	*Fundação para a Ciência e Tecnologia* (FCT)	–	27
	National Structural funding (COMPETE, PRODEP)	–	2
	ARDITI – *Agência Regional para o Desenvolvimento da Investigação*	–	1
International Funding Agencies/Funding Programs	European Commission (includes H2020, IMI, Erasmus Program)	18	17
	Canadian Institutes of Health Research (Canada)	7	0
	European Regional Development Fund	6	0
	Medical Research Council (UK)	7	2
	Australian National Health Service and Medical Research Council	0	5
	National Institutes of Health (NIH, USA)	0	3
	EEA Grants	0	1
	Carlos III Institute of Health (Spain)	0	2

On the other hand, among the 60 IICTs in Portugal receiving financial support from funding grant agencies, only half of these (50%, *n* = 30/60) were funded by national funding grant agencies or funding programs (Fig. 3b) while the other half was funded by international funding agencies. Among the 27 trials funded by *Fundação para a Ciência e Tecnologia* (Table 1) only one (4%, *n* = 1/27) is testing medicinal products (data not shown). During the time period studied in the present work, FCT launched only one call, in 2007, specifically focused in clinical research. The Ministry of Health had a financial contribution to this call and a total amount of 9 M € were used to fund 63 projects [7]. However, none of these funded studies were found in our search, probably because most of them were not interventional studies or interventional without IMPs which registration is desirable but not mandatory. Nevertheless, screening the information provided by FCT we found at least one interventional study with IMP that was not properly classified, consequently not registered in a database by the investigators [8].

The level of participation of Czech Republic and Portugal in international grants was similar (*n* = 38 and *n* = 30, respectively), most of these involving trials with IMPs in both cases (Fig. 3a, b). The organizations receiving national funds, thus acting as sponsor, in higher number of trials in the Czech Republic were hospitals (32%: *n* = 35/109) and universities (22%: *n* = 24/109). Whereas in Portugal were the universities (40%: *n* = 24/60) that sponsored more trials funded by national funding agencies followed by hospitals (7%: *n* = 4/60). Portuguese hospitals sponsored more trials funded by international funding grants then with national funding grant agencies (*n* = 8 vs *n* = 4).

Table 1 shows all the funding grant agencies identified both in the registries and in the publications for each completed and published clinical trial. In the Czech Republic there were a higher number of trials funded by national funding agencies and the number of registrations is gradually growing every year since 2004 (S1, Fig. 1d–e). When compared to the Czech Republic, a lower number of funded IICTs by public funding agencies were identified in Portugal, however, the national investigators have been involved in trials with more diverse funding opportunities, especially international ones.

In the Czech Republic, University hospitals are pressured to apply to grant support and carry out clinical research, because most physicians working in the hospital are university employees at the same time and need to build up their career as researchers. Moreover, there is a lack of experienced grant offices in the Czech Republic which would help the investigators to prepare an application for international grant call, e.g. H2020, IMI2. However, the mandatory insurance to initiate a clinical trial with medicines is not eligible in these grants´ call which might hamper the initiation of more trials. In theory, most Hospitals in Portugal have an office dedicated to support clinical research (as a government decision from 2015) [9]—but only a few can support physicians in the design or implementation of more complex clinical trials involving IMPs or medical devices, or even provide support to prepare proposals for national or international funding. The concept of academic clinical trial units (CTU) introduced in these two countries by ECRIN has been valuable to support clinical investigators and multinational IICTs [9], in the conduct of IICTs, especially in IMPs and/or medical devices trials, during all phases of the process (from the idea until the close out). Universities and biomedical research institutes in Portugal detected earlier the need of professional supporting offices to prepare and manage national and international research projects.

### National Policies for Clinical Research

Both countries present national public policies implemented (or not) for clinical research, in particularly IICTs, which are summarized in Table 2. The data were collected from national legal decisions, analysis of the call’s eligibility criteria for funding national infrastructures and projects as well as higher education institutions in each country.

**Table 2:** National Public Policies Implemented by the Portuguese and Czech Governments Involved in or to Promote Clinical Research.

Public Policies	Czech Republic	Portugal
Establish clinical research as a priority	Yes [10]	Yes [11–13]
Be member of ECRIN-ERIC	Yes [14]	Yes [13]
Define a legal specific status for clinical investigators	Yes [15]	Yes [11]
Investment in public infrastructures to promote/support IICTs	Yes	No
Specific and periodic funding for IICT	Yes	No
National funding agency specific for health research	Yes	No^a^
National public initiatives for clinical research team’s capacitation	No^b^	Yes [16, 17]

*ECRIN*-*ERIC* European Clinical Research Infrastructure Network-European Research Infrastructure Consortium

^a^A national agency (AICIB) for clinical research funding is now implemented in Portugal [18]

^b^A course dedicated to clinical trials management is under preparation

There have been legal decisions to establish clinical research as a priority that included the membership of ECRIN-ERIC and the definition of the clinical investigator status in both countries [10–15]. Since 2015, ten trials were initiated by ECRIN and conducted in the Czech Republic in this time period: POEM_H2020, FAIRPARK II_H2020, NISCI_H2020, SECURE_H2020, EuroHYP_7FP, PrOOf_H2020, VISION_DMD_H2020, DISCHARGE_FP7, PopART_H2020, TENSION_H2020. Some of these projects were also conducted in Portugal (FAIR PARK II, DISCHARGE and POPART). Besides these, the projects MACUSTAR_IMI, BETA3LVH_H2020 and HCQ4Surfedefect_ERA-NET, were also conducted in Portugal making a total of 6 trials initiated in the country through ECRIN. In both countries, none of these trials were from the initiative of national investigators.

The Internal Grant Agency (IGA) of the Ministry of Health supporting medical research and development was established in 1990 in Czech Republic and has been replaced by Czech health research council, AZV, founded on 01/04/2014 [19, 20]. This council, launch calls each year and supports the selected applications of clinical research in the different medicine areas. The establishing the AZV and defining the national priorities of oriented research, experimental development and innovations are the long-term strategies of the Czech government to support the clinical research in medicine. Nevertheless, to our best knowledge in the Czech Republic, the higher education courses specific to train clinical trials professionals are being prepared. In Portugal, the national government supported and invested in specific training for medical doctors^14^ and research team professionals from hospitals [16].

The investment of the Portuguese government, public and private universities in post-graduation courses, including the Portugal Clinical Scholars Research Training (PTCSRT) [17] and the Clinical Investigator Certificate, CLIC [16] is an asset to trigger a high-quality clinical investigation. However, strategies to allocate research time to clinical investigators still need to be implemented [11].

To share knowledge and resources in 2016 a Portuguese government decision created the concept of Clinical Academic Centers (CAC)—joining hospitals, universities and research institutes around a medical school [13]. Currently, 9 CACs are officially established in Portugal, however, they are still deciding on the respective organizational strategy. In line with what is established in the Czech Republic since 2004, in Portugal a new agency to fund exclusively clinical research studies: *Agência para a Investigação Clínica e Investigação Biomédica* (AICIB), focusing on translational medicine products trials, was launched in 2018 but is not yet operational [18].

### Clinical Trials Published in Peer-Reviewed Scientific Journals

**Figure 4:**
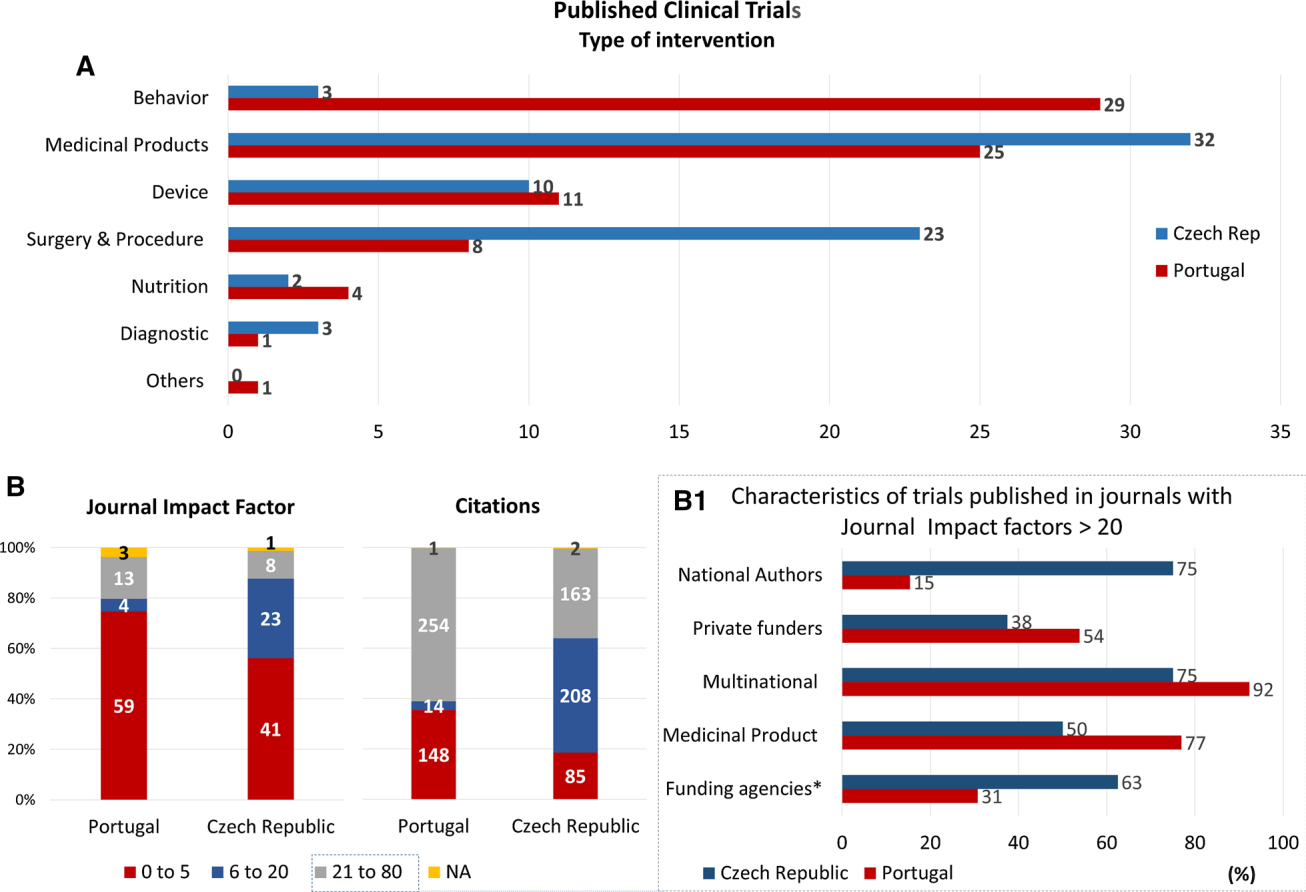
IICTs Published in Peer-Reviewed Journals According to the Type of Intervention (**a**) and Impact Factor of the Journals and Paper Citations Up to 1 Year After Publication (**b**). Papers published in 2018 were not considered. Inset of B (B1): clinical trials published in journals with impact factor higher than 21. * Includes national and international funding agencies.

In the Czech Republic, there are 41% of published trials among the 180 completed (*n* = 73/180) while in Portugal, from the 144 completed trials, 55% (*n* = 79/144) are published in peer-reviewed journals (Fig. 1, step 7). In the Czech Republic, a higher percentage of published studies focus on IMPs (44%, *n* = 32/73), followed by surgery and procedures (32%, *n* = 23/73). On the other hand, in Portugal, most publications focus on behavior studies (37%, *n* = 29/79) followed by IMPs (32%, *n* = 25/79) (Fig. 4a).

The impact factor (IF) of the journal where these IICTs were published varies widely between 0 to 72 as shown in Fig. 4b. Thirty percent (30%, *n* = 23/73) of the trials performed in the Czech Republic with publications were published in journals with IF between 6 to 20. Around 75% (*n* = 59/79) of published papers with trials involving Portuguese organizations, have an IF below 5 and only 16% (*n* = 13/79) were published in journals with IF above 20. The average IF of all these journals is similar for both countries and is around 9. The total number of citations of papers published in Czech Republic and in Portugal up to 1 year after publication were, 458 and 417, respectively (Fig. 4b).

Portuguese investigators are authors/co-authors of 15% (*n* = 2/13) of the papers published in journals with IF > 20, conversely to what is observed with Czech investigators who authored/co-authored of 75% (*n* = 6/8) of the papers published in high impact factor journals (Inset B1, Fig. 4b). Furthermore, international funding agencies funded 50% (*n* = 4/8) of trials in the Czech Republic and only 31% (*n* = 4/13) in Portugal that were published in journals with IF > 21 (Inset B1, Fig. 4b). None of these published trials in high impact journals was funded by the Portuguese national funding agency while in the Czech Republic, one of these trials was funded by the national funding agency. Around half of the trials published in journals with IF > 21 are funded by private funders in Portugal (54%, *n* = 7/13) and around 38% (*n* = 3/8) in the Czech Republic. Additionally, most of these published trials were multinational and focused in IMPs, in both countries.

According to our results the Czech Republic might have had a better return of the investment in this field, publishing a higher number of publications in journals with IF above 6 compared with Portugal (*n* = 32 vs *n* = 20), with a higher number of citations when compared to those published in Portugal. When compared to the Czech Republic, Portugal has slightly a higher number of published completed trials and a higher number of publications in journals with IF greater than 21 (*n* = 13 vs *n* = 8), but the participation of Portuguese recruiting sites does not correspond to more authorships of Portuguese investigators in the publication since only 15% of these publications have Portuguese authors, whereas 75% of publications with the involvement of the Czech Republic recruiting sites have national investigators as authors. The values above mentioned might be underestimated because in this work, only studies registered in CTR and the first publication with results of each completed IICTs were considered.

## Discussion

In this study, a systematic search of IICTs registered in four CTRs was conducted with the main goal of comparing the type of studies, main funders and scientific output in the Czech Republic and in Portugal. The methodology used in this work, allowed us for the first time to have a picture of IICTs in two European countries with similar GDP and population, HDI and number of medical schools, collecting information both from CTRs and publications.

Globally we found a better performance of the Czech Republic in terms of higher number of IICTs, including those with IMPs, of papers published in higher impact factor’s journals, citations, and publication with more national authorships, when compared with Portugal. These findings suggest that a funding agency more focused in clinical research as the Czech Republic funding agency, may facilitate the authorship of national investigators in clinical research studies with higher quality.

In both countries, only a small percentage of the national organizations were sponsoring multinational IICTs in the Czech Republic (6%) and in Portugal (4%), in line with previous reports, where only 3% of all IICTs in the International Clinical Trials Registry Platform (ICTRP) are international [21]. The existence of national funding grant agencies is of major relevance to implement pilot IICTs that later could be escalated to the European level.

The national governments and European Commission investment in clinical research infrastructures have been critical for the development of this area [22, 23]. Both, the Czech Republic and Portugal governments have been investing in European infrastructures of clinical research such as ECRIN-ERIC. However, while in the Czech Republic the government also invested in the national network of CTUs, in Portugal the investment was limited to the ECRIN-ERIC membership fee. Recently a positive discrimination for clinical research has been introduced in the Portuguese infrastructures’ roadmap national strategy [24]. As referred in a previous publication by the same authors, Germany, France, and the UK are good examples to show the increase in the number of IICTs after the investment of respective governments in both the funding of IICTs and supporting infrastructures [4].

When compared to other European countries, Czech Republic and Portugal are below the average both in the number of IICTs/million of citizens and % IICT/total CT [4]. Considering the recent governmental strategies it is clear that this is a work in progress, worthwhile to follow and assess in upcoming years. It would be worth following the example of countries like Denmark (Innovation Fund Denmark) [25] or the UK [26] who establish new rules to support the funding of multinational clinical trials by their national grant agencies. On the other hand, the recent governmental initiatives in Czech Republic and in Portugal to stimulate clinical research might serve as an example for those countries with lower number of IICTs identified in previous publications such as Slovakia and Poland [4, 21]. Additionally, if other countries publish their report assessment on clinical research performance it would facilitate the comparison and extrapolation among European countries policies and outcomes.

The relevance and impact of IICTs for the continuous improvement of therapies and the development of new public policies rely on the results’ independence from the interests of any other stakeholders [27]. Therefore, it is critical for the countries to promote IICTs through a comprehensive and stimulating set of public policies and funding. For example, Italian government decided to invest on IICTs funding, through the Italian Medicines Agency (AIFA) that resulted in an increase of the number of independent clinical research and scientific outputs [28, 29]. This initiative started in 2005 and an amount of 40 M€/year have been provided by the industry to AIFA, to fund competitive IICTs projects developed by Italian physicians working in the public/non-profit sector [29].

As a limitation of this study, the number of IICTs with IMPs in both countries might be underestimated due to lack of registration and/or misclassification, although being mandatory [30, 31]. Similar underestimation might also have occurred in the registration of the other type of interventional studies since these are not mandatory in both countries. Nevertheless, with this work, we expect to reinforce the need of registration of all types of clinical studies, in particularly those funded by national funding agencies. This would facilitate the tracking of the studies in which a huge investment was made, as well as the assessment of the outcomes in terms of publications, guidelines, etc.

In our view, the development of clinical research relies in a national strategy that gather health authorities with science, innovation and economy stakeholders. A change in the way health care units’ value clinical research outcomes and how their performance is evaluated and rewarded, requires a new paradigm. We expect that this work has unveiled the need to explore further details on the IICT quality and funding in other European countries and to learn with those that might have implemented effective indicators to monitor their performance in this particular type of research.

## Conclusions

For the first time, two European Countries were compared in terms of the number of IICTs, funders and the respective scientific outcome. Our results showed that in the same timeframe, the Czech Republic has higher number of registered IICTs and higher number of publications with Czech authors in top journals (impact factors > 21) when compared to Portugal. These findings might be related to the existence of specific and periodic funding for clinical research in the Czech Republic, although the factors behind this difference are difficult to unravel with the methodological approach in this study. We anticipate using the results of this study as a baseline to the better appraisal of IICTs evolution in the upcoming years and inspire other countries to do the same type of evaluation.

## Supplementary Information

Below is the link to the electronic supplementary material.

Electronic supplementary material 1 (DOCX 878 kb) **S1 Fig 1 – Number of IICTs in the Czech Republic and in Portugal** according to the type of intervention (A, A1), therapeutic areas (B,C) and type of funding agency by starting year (D,E).

Electronic supplementary material 2 (xlsx 111 kb) **S2 – Link to access the database**

## Data Availability

All data generated or analyzed during this study are included in this published article [and its supplementary information files].

## Abbreviations

AICIB: *Agência para a Investigação Clínica e Investigação Biomédica*
AIFA: Italian Medicines Agency
ANZCTR: Australian New Zealand Clinical Trials Registry
AZV: Czech health research council
CAC: Clinical Academic Centers
CLIC: Clinical Investigator Certificate
CTR: Clinical Trial Registry
CzeCRIN: Czech Clinical Research Infrastructure Network
ECRIN-ERIC: European Clinical Research Infrastructure Network-European Research Infrastructure Consortium
EU-CTR: European Clinical Trials Registry
FCT: *Fundação para a Ciência e Tecnologia*
GDP: Gross Domestic Product
IICTs: Investigator Initiated Clinical Trials
IMP: Investigational Medicinal Product
ISRCTN: International Standard Randomised Controlled Trial Number
NIH: U.S. National Institutes of Health
PtCRIN: Portuguese Clinical Research Infrastructure Network
PTCSRT: Portugal Clinical Scholars Research Training
