# Supplementary material for: Investigator Initiated Clinical Trials (IICTs): A Systematic Search in Registries to Compare the Czech Republic and Portugal in Terms of Funding Policies and Scientific Outcomes
Source: Ther Innov Regul Sci. 2021 May 18;55(5):966–78. doi: 10.1007/s43441-021-00293-w (PMC8332583; doi:10.1007/s43441-021-00293-w)
Supplement: Supplementary file 1 — Electronic supplementary material 1 (DOCX 878 kb) S1 Fig 1 – Number of IICTs in the Czech Republic and in Portugal according to the type of intervention (A, A1), therapeutic areas (B,C) and type of funding agency by starting year (D,E). [file 43441_2021_293_MOESM1_ESM.docx]

**SUPPLEMENTARY INFORMATION 1**


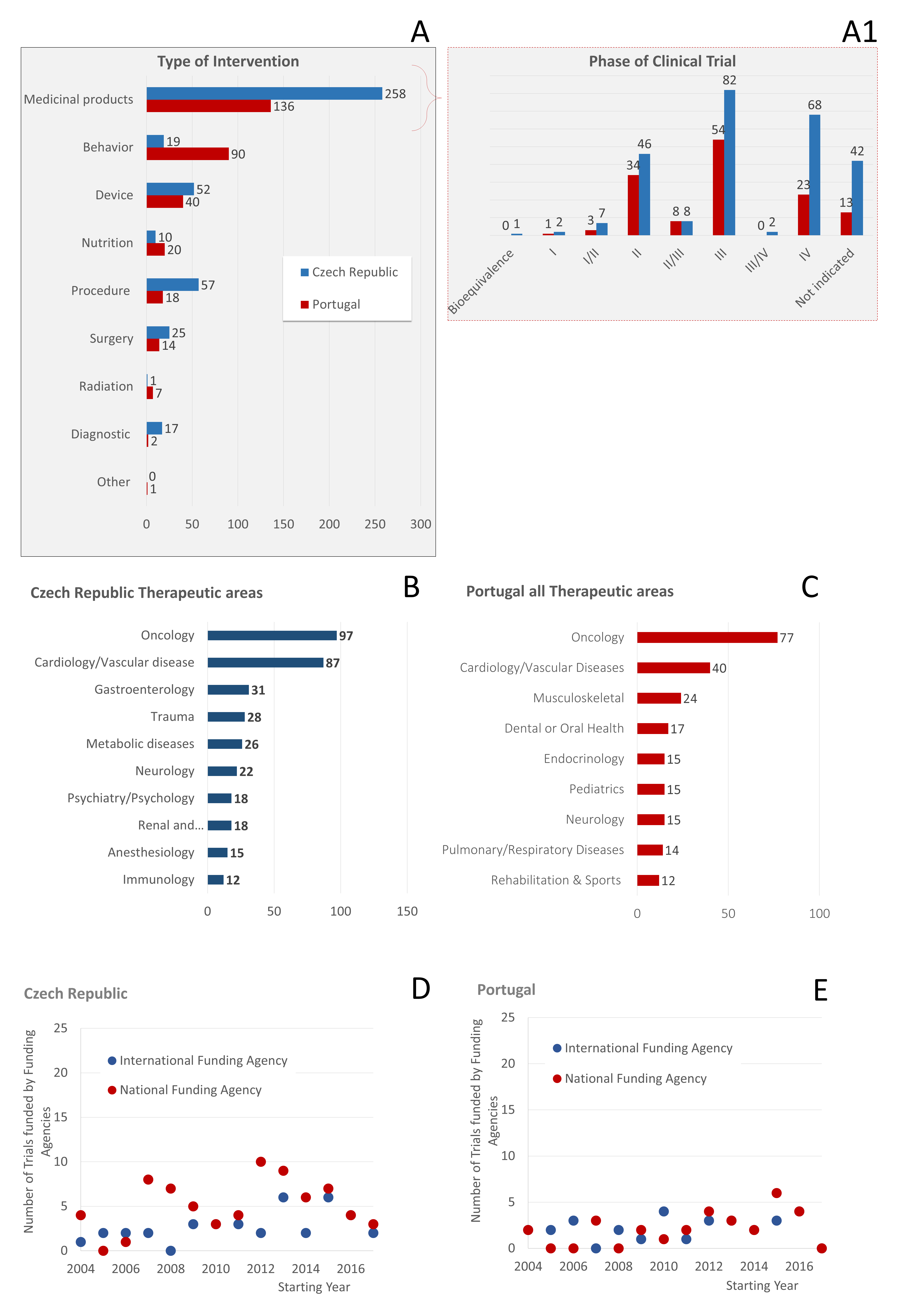


**Figure 1 – Number of IICTs in the Czech Republic and in Portugal** according to the type of intervention (A, A1), therapeutic areas (B,C) and type of funding agency by starting year (D,E).
